# Sleep disorders in children with attention-deficit hyperactivity disorder

**DOI:** 10.4103/0019-5545.55958

**Published:** 2005

**Authors:** Subhash C. Bhargava, Sujata Sethi

**Affiliations:** * Associate Professor, Department of Psychiatry, Pt. B.D. Sharma PGIMS, Rohtak 124001; ** Lecturer, Department of Psychiatry, Pt. B.D. Sharma PGIMS, Rohtak 124001

**Keywords:** Attention-deficit hyperactivity disorder (ADHD), sleep-related involuntary movements, parasomnias

## Abstract

**Background:**

Sleep disturbances are frequently associated with attention-deficit hyperactivity disorder (ADHD) though they are not included in the current classification systems such as the DSM-IV and ICD-10. These problems may complicate the course of the illness as they may be associated with the treatment given

**Aim:**

To evaluate children with ADHD for sleep-related problems.

**Methods:**

The study group comprised 32 children with ADHD and their 20 healthy siblings made up the control group. Sleep-related problems were assessed on a checklist prepared on the basis of the Children Sleep Questionnaire-parent version.

**Results:**

A majority of the children with ADHD had at least one sleep-related problem. Comparison with healthy siblings revealed non-significant differences on the parameters of sleep-related movement disorders and parasomnias.

**Conclusion:**

There is a need for more detailed studies involving sensitive parameters.

## INTRODUCTION

Attention-deficit hyperactivity disorder (ADHD) is the most common problem seen at clinics offering child mental health services and affects approximately 5% of schoolgoing children.^1^ Inattention, impulsiveness and restlessness are described as the core symptoms of ADHD by major classification systems such as the DSM-IV and ICD-10. Other problems such as sleep disturbances, academic under-achievement and poor social relations are not included in the diagnostic criteria. There has been little empirical research on the prevalence and types of sleep problems in ADHD, and their significance remains undocumented.^2^ Most research on sleep in ADHD has focused on parasomnias and sleep-related involuntary movements. The core symptoms of ADHD such as inattention, difficulty in regulating behaviour and emotions, and hyperactivity are strikingly similar to the difficulties caused by disrupted sleep and sleep deprivation.^3^,^4^ Disrupted or shortened sleep has been associated with ADHD-like symptoms.^5^,^6^ The parents of children with ADHD have reported sleep disturbances in their children.^7^,^8^ Studies using the objective measures of sleep, however, have not found clear evidence to support the subjective reports of sleep disturbance in ADHD.^9^,^10^ In a recent review, Corkum *et al*.^2^ examined 16 studies and pointed out the inconsistent nature of the findings of the studies that used objective measures. They opined that these studies suffered from a small sample size, inconsistent diagnostic criteria, lack of exclusion criteria, inadequate control, heterogeneity of sleep parameters, difficulty in adapting to the procedures for assessment in a sleep laboratory. Apart from these, laboratory studies are carried out over only 1 or 2 nights and the child is usually removed from his natural sleeping place. In India, sleep monitoring laboratories are rare. In studies involving psychiatric patients, we found that the reports of the patients and a key informant were fairly reliable and valid. Corkum *et al*.^2^ also report that parents are more likely to report sleep problems on the basis of problematic nights intermixed with better nights; on the other hand, studies with objective criteria are more likely to average and thus miss the pendulum effect. With this aspect in mind, we evaluated children with ADHD for sleep-related problems.

## METHODS

Thirty-two children attending the child guidance clinic at the Department of Psychiatry, Pt B.D. Sharma PGIMS, who met the DSM-IV criteria for ADHD constituted the study sample. All of them were drug-naive. Children who had co-morbid organic disorder were excluded from the study. Twenty healthy siblings of the patients comprised the control group. The children were clinically assessed. Both the parents of each child were interviewed. Sleep-related problems were assessed on a checklist prepared on the basis of the Children Sleep Questionnaire-parent version.^11^

## RESULTS

At least one sleep-related problem was present in 65.62% of children in the ADHD group and in 30% in the sibling group. The total sleep duration of children with ADHD differed only marginally from their healthy siblings. The average sleep duration for the ADHD group was 9 hours and 48 minutes as compared to their healthy siblings in whom the average sleep duration was 10 hours and 28 minutes (Table 1). There was a significant difference in the sleep onset time and awakening time of the two groups (chi-square 6.03 and 6.77, respectively), with more children with ADHD having delayed sleep onset and delayed awakening. On the measures of sleep-related involuntary movements the two groups did not statistically differ from each other though more children with ADHD had sleep-related involuntary movements. Measures of parasomnias, such as night terrors and nightmares, in the two groups could not be compared due to insufficient data for statistical tests.

**Table 1:** Comparison of sleep disorders in children with ADHD and their healthy siblings

Variable	ADHD	Healthy siblings	Chi-square
Sleep duration (hours)	9.48	10.28	
Delayed sleep onset (*n*)	21	6	6.03*
Delayed awakening (*n*)	18	5	6.77*
Restless sleep (*n*)	7	2	1.21
Jerky movements (*n*)	3	—	—
Teeth grinding (*n*)	4	2	0.07
Sleep talking (*n*)	2	2	—
Night terrors (*n*)	2	—	—
Nightmares (*n*)	1	—	—

* p<0.001

## DISCUSSION

The present study compared the sleep pattern of children with ADHD and their healthy siblings. Children with ADHD experienced more sleep-related problems than their healthy siblings. Earlier, Ring *et al*.^12^ also reported that significantly more children with ADHD reported single or multiple sleep disturbances as compared to their siblings. Children with ADHD experienced problems mainly in the area of dyssomnias. They had delayed sleep onset as well as delayed awakening. Owens *et al*.^13^ reported shorter sleep duration and more disturbed sleep. Stein^14^ reported increased sleep latency but this was more in the group treated with stimulants. The two groups could not be compared on the measures of parasomnias as nightmares and night terrors were reported only in the ADHD group. Corkum *et al*.,^15^ studying children with ADHD, reported no difference in parasomnias in the clinical and non-clinical groups. The results of this preliminary investigation suggest a relationship between sleep disturbances and ADHD, and support the finding that an unstable sleep–wake system is characteristic of children with ADHD.

## CONCLUSION

Sleep-related problems may have a significant bearing on the course and management of ADHD. A careful evaluation of sleep history is recommended in children with ADHD. Further research using sensitive tools is needed to evaluate the relationship between ADHD and sleep-related problems.
